# Predictive Performance of SAPS-3, SOFA Score, and Procalcitonin for Hospital Mortality in COVID-19 Viral Sepsis: A Cohort Study

**DOI:** 10.3390/life15081161

**Published:** 2025-07-23

**Authors:** Roberta Muriel Longo Roepke, Helena Baracat Lapenta Janzantti, Marina Betschart Cantamessa, Luana Fernandes Machado, Graziela Denardin Luckemeyer, Joelma Villafanha Gandolfi, Bruno Adler Maccagnan Pinheiro Besen, Suzana Margareth Lobo

**Affiliations:** 1 Trauma and Acute Care Surgery ICU, Hospital das Clínicas (HCFMUSP), Faculdade de Medicina, Universidade de São Paulo, São Paulo 05403-010, SP, Brazil; 2 Division of Intensive Care Medicine, Hospital de Base, Faculdade de Medicina de São José do Rio Preto (FAMERP), Av. Brigadeiro Faria Lima 5544, Vila São José, São José do Rio Preto 15090-000, SP, Brazil; 3 Postgraduate Program in Medical Sciences, Faculdade de Medicina, Universidade de São Paulo, São Paulo 01246-903, SP, Brazil; 4 IDOR Research and Education Institute, São Paulo 01401-002, SP, Brazil; 5 Graduate Program in Health Sciences, Faculdade de Medicina de São José do Rio Preto (FAMERP), São Paulo 15090-000, SP, Brazil

**Keywords:** ORGAN disfunction score, procalcitonin, C-reactive protein, prognosis, biomarkers, predictive value of test, sepsis, COVID-19

## Abstract

**Objective:** To evaluate the prognostic utility of the Sequential Organ Failure Assessment (SOFA) and Simplified Acute Physiology Score 3 (SAPS 3) in COVID-19 patients and assess whether incorporating C-reactive protein (CRP), procalcitonin, lactate, and lactate dehydrogenase (LDH) enhances their predictive accuracy. **Methods:** Single-center, observational, cohort study. We analyzed a database of adult ICU patients with severe or critical COVID-19 treated at a large academic center. We used binary logistic regression for all analyses. We assessed the predictive performance of SAPS 3 and SOFA scores within 24 h of admission, individually and in combination with serum lactate, LDH, CRP, and procalcitonin. We examined the independent association of these biomarkers with hospital mortality. We evaluated discrimination using the C-statistic and determined clinical utility with decision curve analysis. **Results:** We included 1395 patients, 66% of whom required mechanical ventilation, and 59.7% needed vasopressor support. Patients who died (39.7%) were significantly older (61.1 ± 15.9 years vs. 50.1 ± 14.5 years, *p* < 0.001) and had more comorbidities than survivors. Among the biomarkers, only procalcitonin was independently associated with higher mortality in the multivariable analysis, in a non-linear pattern. The AUROC for predicting hospital mortality was 0.771 (95% CI: 0.746–0.797) for SAPS 3 and 0.781 (95% CI: 0.756–0.805) for the SOFA score. A model incorporating the SOFA score, age, and procalcitonin demonstrated high AUROC of 0.837 (95% CI: 0.816–0.859). These associations with the SOFA score showed greater clinical utility. **Conclusions:** The SOFA score may aid clinical decision-making, and incorporating procalcitonin and age could further enhance its prognostic utility.

## 1. Introduction

Prognostic models are routinely used tools to predict outcomes in critically ill patients, particularly for benchmarking. The Simplified Acute Physiology Score 3 (SAPS 3) is a widely used prognostic score that assesses illness severity and estimates hospital mortality risk [1]. In contrast, organ dysfunction scores, such as the Sequential Organ Failure Assessment (SOFA) score, were originally developed to quantify organ dysfunction and failure in sepsis patients [2]. Over time, the SOFA score has been shown to strongly correlate with mortality across a broad population of critically ill patients. The SAPS 3 can also be used to monitor ICU performance over time and for benchmarking purposes. However, the SOFA score is still widely used to compare different ICUs regarding their burden of acute organ dysfunction [3,4].

These models for diagnosing or forecasting clinical outcomes have gained increasing importance in the era of precision medicine, but external validation is seldom performed. Efforts to externally validate models are necessary to test their performance on a different sample from the one used for their development. For example, the SOFA score demonstrated superior predictive performance for hospital mortality in critically ill trauma patients compared to anatomical injury-based scores such as the Injury Severity Score (ISS) and the New Injury Severity Score (NISS) [5]. In sepsis, a modified cardiovascular SOFA score (CV SOFA score) demonstrated slightly better calibration compared to the original cardiovascular sub-score of the SOFA score [6]. In COVID-19, both SAPS 3 and SOFA scores were widely used for population characterization and benchmarking [6]. The SOFA score was a significant predictor of mortality in critically ill COVID-19 patients; however, a small study suggested that initial SOFA scores may not be reliable for prognostication [7,8].

Additionally, elevated serum levels of biomarkers such as C-reactive protein (CRP) and procalcitonin (PCT) have been associated with worse outcomes in critically ill patients requiring intensive care [9,10,11]. Other biomarkers, such as lactate dehydrogenase (LDH), a biomarker of pneumocyte injury, and lactate, a biomarker of cellular metabolic stress, are also commonly used and widely available in clinical practice, but their predictive ability over and beyond traditional organ dysfunction scores in viral sepsis is not fully understood [12]. Moreover, older age has been linked to poorer outcomes in COVID-19 patients [13]. However, the incremental prognostic value of these biomarkers beyond established prognostic and organ dysfunction scores remains insufficiently studied.

In this study, we aimed to evaluate the predictive performance of two widely used prognostic and organ dysfunction scores in critically ill COVID-19 patients admitted to the ICU, as well as the added prognostic value of lactate, CRP, PCT, and LDH.

## 2. Methods

### 2.1. Study Design, Setting, and Ethics

This is a single-center cohort study conducted at Hospital de Base in São José do Rio Preto, São Paulo, Brazil; a 900-bed tertiary university hospital and designated COVID-19 treatment center with 40 ICU beds. We performed a retrospective analysis of prospectively collected data from critically ill COVID-19 patients. The local Institutional Review Board (CAAE: 31715710.1.0000.5415) approved the study and waived the need for informed consent due to its retrospective design. We reported this study according to the Transparent Reporting of a Multivariable Prediction Model for Individual Prognosis or Diagnosis (TRIPOD) guidelines for validation studies [14].

### 2.2. Inclusion and Exclusion Criteria

We screened patients admitted to the ICU between March 2020 and November 2021 who were over 18 years old, were confirmed to have SARS-CoV-2 infection via a positive polymerase chain reaction (PCR) test from a nasopharyngeal sample and met the criteria for severe or critical disease. We excluded patients who tested PCR negative, readmissions or who were discharged in less than 48 hours. Severe COVID-19 cases were defined by clinical signs of pneumonia and at least one of the following: respiratory rate > 30 breaths per minute, severe respiratory distress, and/or oxygen saturation (SpO1) < 90% on room air. Critical COVID-19 cases were those with acute respiratory distress syndrome or respiratory failure requiring supplementary oxygen therapy, non-invasive or invasive ventilation, sepsis, or septic shock [15].

### 2.3. Outcome

We assessed predictive performance with hospital mortality as our main outcome.

### 2.4. Data Collection and Variables

Trained physicians prospectively collected data on demographic characteristics, comorbidities, clinical information, laboratory tests, and the type and duration of organ support (mechanical ventilation, dialysis, vasoactive drugs). SAPS 3 was calculated upon ICU admission, while the SOFA score was recorded during the first three days of ICU stay [1,2]. Among other routinely collected laboratory tests, we analyzed serum lactate (a biomarker of cellular dysfunction), lactate dehydrogenase (LDH, a marker of pneumocyte injury), C-reactive protein (CRP, an indicator of inflammation), and procalcitonin (PCT, a marker of inflammation and potential bacterial infection).

### 2.5. Statistical Analysis

We selected a sample of patients admitted during the study period, ensuring there were a sufficient number of events and non-events to adequately assess our objective. For the descriptive analysis, we presented categorical variables as absolute frequencies and percentages, and continuous variables as means with standard deviations or medians with corresponding 25th and 75th percentiles, depending on their distribution. We used the chi-squared test to compare categorical variables and t-tests or Mann-Whitney tests for quantitative variables, as appropriate. We assessed the distribution of the biomarkers and the SOFA score with histograms to evaluate how to assess them in the models.

To assess the biomarkers’ prognostic associations with hospital mortality, we used logistic regression and included the variables in the model as restricted cubic splines to allow for the evaluation of non-linear associations, which are presented graphically. We first performed an unadjusted analysis, followed by an analysis adjusted for SAPS 3, both in the complete case analysis sample. We evaluated discrimination using the C-statistic and by comparing the areas under two or more receiver operating characteristic (ROC) curves [16].

To assess the discrimination of the SAPS 3 model, SOFA score, and biomarkers, we used a multiply imputed dataset. We considered the data to be missing at random (Supplemental File, Table S1). It consisted of 50 imputations, which included the outcome, the variables to be included in the model (in their functional format, as splines), and auxiliary variables (such as use of vasoactive drugs, mechanical ventilation and renal replacement therapy). Imputations were performed with logistic regression (for categorical variables) or predictive mean matching (for continuous variables) [17]. We then assessed discrimination, apparent calibration (through loess plots of predicted vs. observed probabilities) [18], and the net benefit of the SAPS 3 model, SOFA score, and different combinations of variables, including the biomarker that was associated with worse outcomes after accounting for other clinically relevant variables. Net benefit is presented as decision curve analysis plots [19]. We used Wald tests to conduct hypothesis tests. *p*-values < 0.05 were considered statistically significant, with no adjustment for multiple comparisons. We analyzed data in Stata SE 18.0.

## 3. Results

From a cohort of 1592 consecutive ICU admissions, there were 197 exclusions due to absent core data, and 1395 patients were enrolled (Figure S1). Table 1 summarizes their main characteristics. The overall mortality rate was 39.7%. The average patient age was 54.8 ± 16.1 years, and 57.9% were male. The mean SOFA score was 5.6 ± 3.8, and the SAPS 3 was 57.7 ± 16.4. Among the patients, 66% required mechanical ventilation, and 59.7% needed vasopressor support. Additional information about data missingness is presented in Table S2.

Non-survivors were significantly older compared to survivors (61.1 ± 15.9 years vs. 50.1 ± 14.5 years, *p* < 0.001) and had more comorbidities, as indicated by a higher Charlson Index score (1 [IQR 0–1] vs. 0 [IQR 0–1], *p* < 0.001). Non-survivors had significantly higher SAPS 3 and SOFA score, as well as elevated serum lactate, CRP, PCT, and LDH levels at admission compared to survivors (Table 1). They also required more intensive ventilatory and vasopressor support for longer durations.

**Figure 1:**
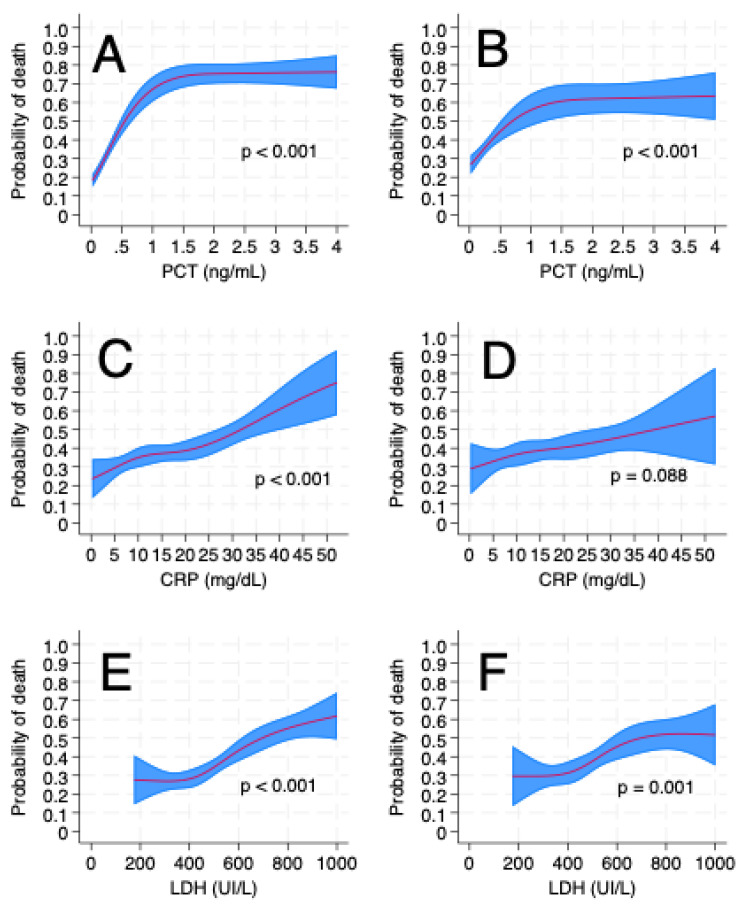

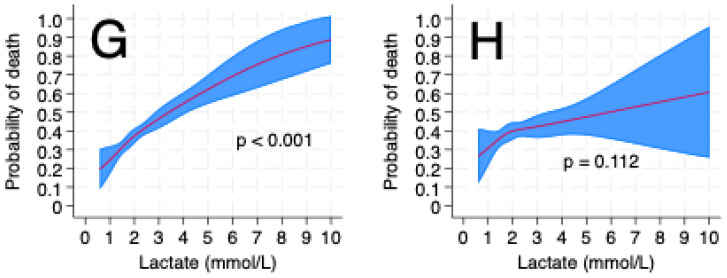
Nonlinear association of biomarkers at ICU admission and mortality. All variables were modeled as restricted cubic splines with 4 knots (lactate, LDH and CRP) or 3 knots (PCT) unadjusted (left column, (**A**,**C**,**E**,**G**)) or adjusted for illness severity with the SAPS 3 (right column, (**B**,**D**,**F**,**H**)). *p*-values were obtained from a Wald test of the joint effect of the restricted cubic splines.

The distribution of the biomarkers is presented in the Supplemental File, stratified by hospital mortality (Supplemental File, Figures S1–S5). The SOFA score presented a near-linear distribution with hospital mortality (Supplemental File, Figure S6). Figure 1 presents the non-linear relationships between serum lactate, DHL, PCR, and PCT at admission and hospital mortality. All variables were significantly associated with hospital mortality in the unadjusted analysis (Figure 1, left column). After adjusting for illness severity by the SAPS 3 (Figure 1, right column), only LDH and PCT remained associated with worse outcomes.

**Figure 2:**
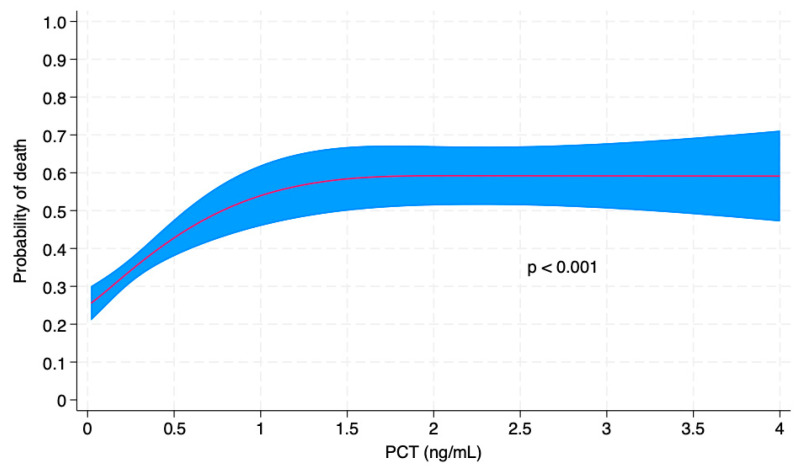
Non-linear association of procalcitonin at ICU admission with hospital mortality, adjusted for SOFA score, age and Charlson comorbidity index. Results were obtained from a multiply imputed dataset, with procalcitonin values truncated at 4 and 3-knot restricted cubic splines. The *p*-value is obtained from a Wald test of the joint effect of the splines.

After adjusting for age, organ dysfunction severity (SOFA score), and comorbidities (Charlson Comorbidity Index), only PCT remained independently associated with worse outcomes (Figure 2).

**Figure 3:**
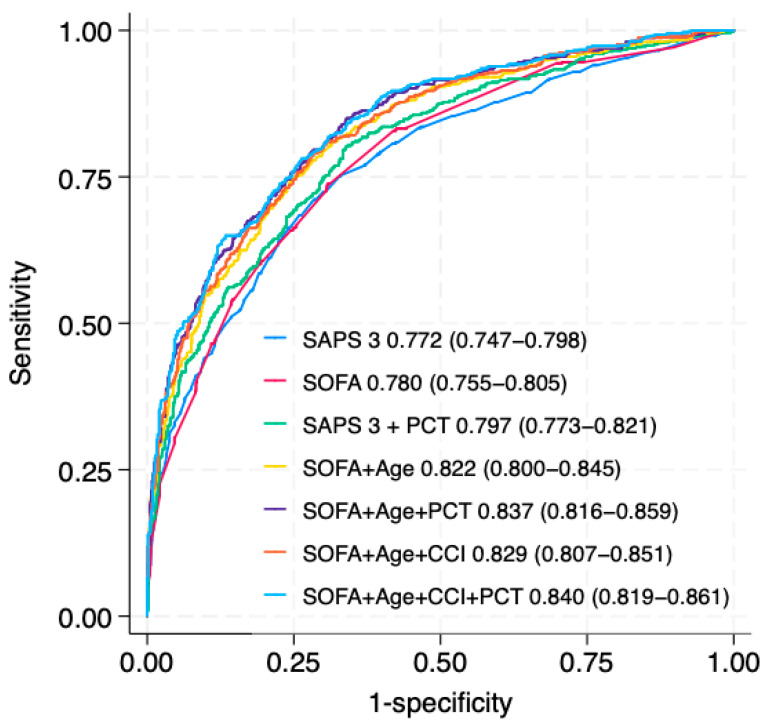
Areas under the receiving operator characteristic curve (AUROCs) for SAPS 3, SOFA score, and other combinations of variables. SAPS 3: simplified acute physiology score, 3rd version; SOFA: sequential organ failure assessment; PCT: procalcitonin; CCI: Charlson comorbidity index.

Figure 3 illustrates the discrimination of SAPS 3, SOFA score, and their respective augmented models in predicting hospital mortality. Adding procalcitonin improved the AUROC for both SAPS 3 and SOFA score. It also enhanced the AUROC when combined with different variations of SOFA scores and other variables. Figure S7 presents the apparent calibration of all models.

**Figure 4:**
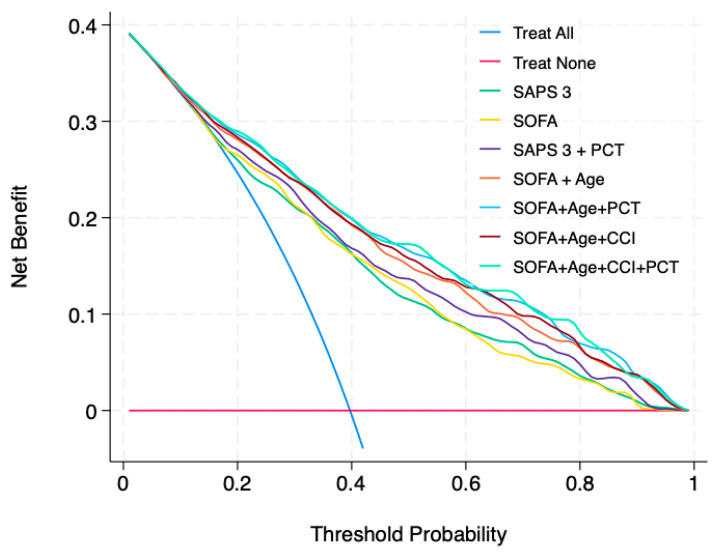
Decision curve analysis of SAPS 3, SOFA score, and added variables to the model. SAPS 3: simplified acute physiology score, 3rd version; SOFA: sequential organ failure assessment; PCT: procalcitonin; CCI: Charlson comorbidity index.

Figure 4 shows the decision curve analysis for SAPS 3, SOFA score, and each combination of variables. While the SAPS 3 and SOFA score demonstrated similar net benefits across different probability thresholds, the combination of the SOFA score with age, as well as the SOFA score with age and comorbidities, outperformed SAPS 3 alone. Adding procalcitonin further increased the net benefit across a wide range of probability thresholds.

## 4. Discussion

### 4.1. Main Findings

Our manuscript highlights several key findings. First, the SOFA score and SAPS 3 exhibited comparable AUROCs for predicting hospital mortality in critically ill COVID-19 patients. Second, among the biomarkers studied, only procalcitonin remained significantly associated, in a non-linear fashion, with hospital mortality after adjusting for either SAPS 3 or a combination of age, SOFA score, and comorbidities. Third, decision curve analysis demonstrated that incorporating the SOFA score, age, comorbidities, and procalcitonin enhances mortality risk prediction in this critically ill population.

### 4.2. Relationship with the Literature

The SOFA score is widely recognized as a key predictor of mortality in critically ill COVID-19 patients. In a study of 375 mechanically ventilated adults, a higher SOFA score on day 1 was strongly associated with ICU mortality. Other contributing factors included the pandemic wave, use of Remdesivir, occurrence of acute kidney injury (AKI), sepsis, enteral insufficiency, ICU length of stay, and white blood cell count [20]. Patients with SOFA scores ≥ 5 had significantly lower survival rates than those with scores < 5 [21]. However, in a larger cohort of 1044 patients with confirmed COVID-19 pneumonia, the SOFA score demonstrated only modest predictive performance. This may be due to the study population not being restricted to ICU patients [22]. Notably, age showed greater predictive accuracy for in-hospital mortality than the SOFA score in COVID-19 patients [23]. Beyond COVID-19, the SOFA score has shown superior predictive value for hospital mortality in critically ill trauma patients, outperforming anatomical injury-based scores such as the ISS and the NISS. In a validation cohort study, the SOFA score achieved an AUROC of 0.807, significantly surpassing both ISS and NISS [5].

CRP is not only a good biomarker of inflammation but also acts as a direct participant in the pathological process. CRP is typically elevated in bacterial infections, but its levels can also rise in severe viral infections, thereby providing insights into the host’s inflammatory status [23]. Although CRP is frequently reported as a prognostic marker, it did not provide additional prognostic value beyond illness severity in this sample of critically ill COVID-19 participants with multiple organ failure. This contrasts with the scenario of undifferentiated COVID-19 patients with a full spectrum of disease recently admitted to the hospital, in which CRP had prognostic value, which led to its inclusion in the 4C Mortality score [24].

While procalcitonin (PCT) is considered more specific for bacterial infections, its levels also correlate with disease severity, making it an unreliable marker for distinguishing between bacterial and nonbacterial infections in critically ill patients, particularly in severe influenza and coronavirus cases [11]. Procalcitonin was the only biomarker with added prognostic information after accounting for illness severity of intensity of organ dysfunction and age. Its relationship with hospital mortality was non-linear, suggesting the need to account for this when using this biomarker at the bedside. Higher PCT levels are associated with increased disease severity and higher mortality rates in COVID-19 patients, but do not predict bacterial coinfection [25]. Several factors may explain why PCT remained the only biomarker independently linked to worse outcomes after adjusting for other relevant variables. Many previous studies failed to adequately account for illness severity or incorporate a comprehensive set of well-established prognostic factors, as we did.

In our cohort, lactate was not independently associated with worse outcomes after adjusting for illness severity, which was possibly due to adjustment for multiple organ dysfunction in our models. Another potential explanation is its pulmonary production [26], which may correlate with respiratory dysfunction, leading to collinearity. Similarly, LDH was not associated with worse outcomes after accounting for organ dysfunction and other variables. Although LDH is a biomarker of pneumocyte injury, its close association with severity in respiratory dysfunction may have diminished its independent prognostic value. Additionally, PCT may have identified patients who are more likely to have bacterial co-infection, which could explain why it remained associated with worse outcomes, reaching a plateau at values above

Beyond discrimination and calibration, decision curve analysis is a valuable tool for assessing model performance by quantifying its clinical utility in decision-making, such as guiding treatment adjustments and improving patient outcomes [27,28]. In our analysis, the SOFA score provided a comparable net benefit to the SAPS 3 score across a broad range of outcome probabilities. Moreover, adding PCT further enhanced the net benefit of the SOFA score.

### 4.3. Implications

Our results should be interpreted in the context of phase 2 prognostic studies [29]. This means that we did not intend to derive a prognostic model (phase 3) [30], nor evaluate its utility for stratified medicine (phase 4) [31]. As a phase 2 prognostic study, we could demonstrate the prognostic utility of SAPS 3 score, which is expected, but more importantly, the similar prognostic utility of the SOFA score, which could be markedly enhanced by including age and procalcitonin as further risk factors for death. As such, these easily available and calculated variables could be used at the bedside to inform shared decision-making. Furthermore, future studies should consider adding PCT to prognostic scores (instead of CRP, for example).

### 4.4. Strengths and Limitations

The strengths of our study include a large sample size and, more importantly, the availability and evaluation of the added benefit of biomarkers over and above a prognostic score and an organ dysfunction score. We also used restricted cubic splines, which allowed the evaluation of non-linearities in the association between the biomarkers and hospital mortality. We also present the decision-curve analysis plots, which enhance clinical interpretation of the results. However, our study has some limitations. Its retrospective nature may introduce biases related to the unavailability of additional data or missing data. Nevertheless, we used multiple imputations to address this issue. The study may not have fully accounted for all potential confounders, such as variations in clinical management. The study focused exclusively on critically ill patients, so the results may not apply to those with milder forms of COVID-19. We also only evaluated the models’ apparent calibration, which may have led to overoptimistic calibration that needs further external validation. Finally, as it is a single-center study, the findings may not be generalizable to all ICUs and, most importantly, they cannot be transported to less severely ill populations, in which mortality scores have been described with good performance.

## 5. Conclusions

In summary, we observed that the SOFA score is a valuable tool for guiding clinical decision-making in critically ill COVID-19 patients, with performance similar to SAPS 3. However, relying solely on the SOFA score appears insufficient to fully characterize disease severity in this population. Additional factors, such as age, other biomarkers, and especially procalcitonin, are essential for a more comprehensive evaluation and enhanced prognostic assessment.

## Figures and Tables

**Table 1:** Study cohort characteristics stratified by survivors and non-survivors.

Variables	All Cohort (N = 1395)	Survivors (n = 841)	Non Survivors (n = 554)	*p* Value
Age, years	54.8 ± 16.1	50.1 ± 14.5	61.1 ± 15.9	<0.001
Sex, male	808 (57.9%)	479 (57%)	319 (59.4%)	0.38
Hypertension (%)	670 (61.2%)^301^	339 (55.2%)^227^	331 (69.0%)^74^	<0.001
Diabetes (%)	396 (36.2%)^301^	187 (30.5%)^227^	209 (43.5%)^74^	<0.001
Obesity (%)	220 (20.2%)^305^	136 (22.3%)^230^	84 (17.5%)^75^	0.057
Coronary heart disease (%)	78 (7.1%)^302^	31 (5.0%)^227^	47 (9.8%)^75^	0.003
Asthma (%)	40 (3.7%)^302^	20 (3.3%)^228^	20 (4.2%)^74^	0.52
COPD (%)	77 (7.0%)^301^	28 (4.6%)^227^	49 (10.2%)^74^	<0.001
Heart failure (%)	38 (3.5%)^301^	15 (2.4%)^227^	23 (4.8%)^74^	0.045
Chronic kidney disease (%)	34 (5.4%)^768^	7 (1.9%)^481^	27 (10.1%)^287^	<0.001
Cirrhosis (%)	15 (1.4%)^302^	6 (1.0%)^228^	9 (1.9%)^74^	0.29
Immunossupression (%)	42 (3.8%)^304^	13 (2.1%)^230^	29 (6.0%)^74^	0.001
CCI	1.0 (0.0–1.0)^191^	0 (0.0–1.0)^101^	1.0 (0.0–1.0)^91^	<0.001
SAPS 3	57.7 (16.4) ^133^	51.5 (11.3)^91^	66.8 (17.4)^41^	<0.001
SOFA	5.6 (3.0–9.0)^154^	3.0 (2.0–5.0)^91^	8.0 (4.0–11.0)^63^	<0.001
Lactate, mEq/L	1.1 (1.6–1.7)^91^	1.0 (1.6–1.5)^51^	1.3 (1.7–1.9)41	<0.001
CRP, mg/dL	14.1 (8.1–11.9)^489^	13.4 (7.5–10.9)^181^	16.1 (9.7–16.1)^108^	<0.001
PCT, ng/mL	0.11 (0.1–0.6)^313^	0.1 (0.1–0.3)^174^	0.5 (0.1–1.5)^139^	<0.001
LDH, U/L	494 (380–632)^638^	459 (366–579)^306^	559.5 (415–691)^151^	<0.001
RRT (%)	135 (17.1)	39 (4.6)	196 (35.4)	<0.001
MV (%)	910 (65.9)	395 (47)	515 (94.8)	<0.001
MV, days	5 (0.0–13.0)	0.0 (0.0–10.0)	9.0 (4.0–17.0)	<0.001
Vasoactive drug (%)	831 (59.7)	344 (40.9)	488 (88.1)	<0.001
ICU LOS, days	11.0 (5.0–19.0)	11.0 (6.0–19.0)	11.0 (5.0–10.0)	0.11
Hospital LOS, days	15.0 (9.0–16.0)	16.0 (11.0–17.0)	13.0 (6.0–14.0)	<0.001

Abbreviations: CCI: Charlson comorbidity index; COPD: chronic obstructive pulmonary disease; SAPS 3: simplified cute physiology score: 3rd version; SOFA: sequential organ failure assessment; CRP: C-reactive protein; PCT: procalcitonin; LDH: lactic dehydrogenase; RRT: renal replacement therapy; MV: mechanical ventilation; ICU: intensive care unit; LOS: length-of-stay. Numbers are presented as mean ± SD, n (%) or median (25th–75th percentiles). ^n^: missing numbers.

## Data Availability

The data that support the findings of this study are not openly available due to restrictions in ethical approval.

## Supplementary Materials

The following supporting information can be downloaded at: https://www.mdpi.com/article/10.3390/life15081161/s1, Figure S1: Lactate histogram stratified by hospital mortality; Figure S2: Lactate dehydrogenase histogram; Figure S3: C-reactive protein histogram; Figure S4: Procalcitonin histogram; Figure S5: Admission SOFA score histogram stratified by hospital mortality; Figure S6: Bar plot of risk of hospital death stratified by admission SOFA score; Figure S7: Apparent calibration for SAPS 3, SOFA score and other combinations of variables; Table S1: Comparison of characteristics between patients with and without missing data; Table S2: *p*-values of the non-linear associa0on (restricted cubic splines) of lactate, CRP, PCT and LDH with hospital mortality after accounting for illness severity (SAPS 3) or relevant covariates (SOFA, age and CCI).

## References

[1] Moreno R.P., Metnitz P.G., Almeida E., Jordan B., Bauer P., Campos R.A., Iapichino G., Edbrooke D., Capuzzo M., Le Gall J.R. (2005). SAPS 3—From evaluation of the patient to evaluation of the intensive care unit. Part 1: Development of a prognostic model for hospital mortality at ICU admission. Intensive Care Med..

[2] Vincent J.L., Moreno R., Takala J., Willatts S., De Mendonça A., Bruining H., Reinhart C.K., Suter P.M., Thijs L.G. (1996). The SOFA (Sepsis-related Organ Failure Assessment) score to describe organ dysfunction/failure. On behalf of the Working Group on Sepsis-Related Problems of the European Society of Intensive Care Medicine. Intensive Care Med..

[3] Pölkki A., Pekkarinen P.T., Takala J., Selander T., Reinikainen M. (2022). Association of Sequential Organ Failure Assessment (SOFA) components with mortality. Acta Anaesthesiol. Scand..

[4] Quintairos A., Rezende E.A.D.C., Soares M., Lobo S.M.A., Salluh J.I.F. (2022). Leveraging a national cloud-based intensive care registry for COVID-19 surveillance, research and case-mix evaluation in Brazil. Rev. Bras. Ter. Intensiv..

[5] Roepke R.M.L., Besen B.A.M.P., Daltro-Oliveira R., Guazzelli R.M., Bassi E., Salluh J.I.F., Damous S.H.B., Utiyama E.M., Malbouisson L.M.S. (2023). Predictive Performance for Hospital Mortality of SAPS 3, SOFA, ISS, and New ISS in Critically Ill Trauma Patients: A Validation Cohort Study. J. Intensive Care Med..

[6] Lee H.J., Ko B.S., Ryoo S.M., Han E., Suh G.J., Choi S.H., Chung S.P., Lim T.H., Kim W.Y., Kwon W.Y. (2022). Modified cardiovascular SOFA score in sepsis: Development and internal and external validation. BMC Med..

[7] Khan M.H., Ali M.A., Salim B. (2024). Prediction of Mortality Using the Sequential Organ Failure Assessment Score in Critically Ill COVID-19 Patients. J. Coll. Physicians Surg. Pak..

[8] Gruyters I., De Ridder T., Bruckers L., Geebelen L., Gharmaoui S., Callebaut I., Vandenbrande J., Berends N., Dubois J., Stessel B. (2022). Predictive value of serial evaluation of the Sequential Organ Failure Assessment (SOFA) score for intensive care unit mortality in critically ill patients with COVID-19: A retrospective cohort study. Anaesthesiol. Intensive Ther..

[9] Póvoa P., Coelho L., Cidade J.P., Ceccato A., Morris A.C., Salluh J., Nobre V., Nseir S., Martin-Loeches I., Lisboa T. (2024). Biomarkers in pulmonary infections: A clinical approach. Ann. Intensive Care.

[10] Moreno M.S., Nietmann H., Matias C.M., Lobo S.M. (2010). C-reactive protein: A tool in the follow-up of nosocomial pneumonia. J. Infect..

[11] Maves R.C., Enwezor C.H. (2022). Uses of Procalcitonin as a Biomarker in Critical Care Medicine. Infect. Dis. Clin..

[12] Ferreira J.C., Ho Y.-L., Besen B.A.M.P., Malbouisson L.M.S., Taniguchi L.U., Mendes P.V., Costa E.L.V., Park M., Daltro-Oliveira R., Roepke R.M.L. (2021). Protective ventilation and outcomes of critically ill patients with COVID-19: A cohort study. Ann. Intensive Care.

[13] Michels E.H., Appelman B., de Brabander J., van Amstel R.B., Chouchane O., van Linge C.C., Schuurman A.R., Reijnders T.D., Sulzer T.A., Klarenbeek A.M. (2023). Age-related changes in plasma biomarkers and their association with mortality in COVID-19. Eur. Respir. J..

[14] Moons K.G., Altman D.G., Reitsma J.B., Collins G.S. (2015). Transparent Reporting of a Multivariate Prediction Model for Individual Prognosis or Development Initiative. New Guideline for the Reporting of Studies Developing, Validating, or Updating a Multivariable Clinical Prediction Model: The TRIPOD Statement. Adv. Anat. Pathol..

[15] Alhazzani W., Møller M.H., Arabi Y.M., Loeb M., Gong M.N., Fan E., Oczkowski S., Levy M.M., Derde L., Dzierba A. (2020). Surviving Sepsis Campaign: Guidelines on the Management of Critically Ill Adults with Coronavirus Disease 2019 (COVID-19). Crit. Care Med..

[16] DeLong E.R., DeLong D.M., Clarke-Pearson D.L. (1988). Comparing the areas under two or more correlated receiver operating characteristic curves: A nonparametric approach. Biometrics.

[17] Van Calster B., McLernon D.J., Van Smeden M., Wynants L., Steyerberg E.W. (2019). Topic Group ‘Evaluating diagnostic tests and prediction models’ of the STRATOS initiative. Calibration: The Achilles heel of predictive analytics. BMC Med..

[18] Vickers A.J., Elkin E.B. (2006). Decision curve analysis: A novel method for evaluating prediction models. Med. Decis. Mak..

[19] Vesin A., Azoulay E., Ruckly S., Vignoud L., Rusinovà K., Benoit D., Soares M., Azeivedo-Maia P., Abroug F., Benbenishty J. (2013). Reporting and handling missing values in clinical studies in intensive care units. Intensive Care Med..

[20] Lavrentieva A., Kaimakamis E., Voutsas V., Bitzani M. (2023). An observational study on factors associated with ICU mortality in Covid-19 patients and critical review of the literature. Sci. Rep..

[21] Gómez-Romero F.J., Muñoz-Rodríguez J.R., Serrano-Oviedo L., García-Jabalera I., López-Juárez P., Pérez-Ortiz J.M., Redondo-Calvo F.J. (2022). COVID-19 SESCAM Network. Clinical features and mortality of COVID-19 patients admitted to ICU according to SOFA score. Medicine.

[22] Luan Y.Y., Yin C.H., Yao Y.M. (2021). Update Advances on C-Reactive Protein in COVID-19 and Other Viral Infections. Front. Immunol..

[23] Vedovati M.C., Barbieri G., Urbini C., D’AGostini E., Vanni S., Papalini C., Pucci G., Cimini L.A., Valentino A., Ghiadoni L. (2022). Clinical prediction models in hospitalized patients with COVID-19: A multicenter cohort study. Respir. Med..

[24] Knight S.R., Ho A., Pius R., Buchan I., Carson G., Drake T.M., Dunning J., Fairfield C.J., Gamble C., Green C.A. (2020). Risk stratification of patients admitted to hospital with covid-19 using the ISARIC WHO Clinical Characterisation Protocol: Development and validation of the 4C Mortality Score. BMJ.

[25] Galli F., Bindo F., Motos A., Fernández-Barat L., Barbeta E., Gabarrús A., Ceccato A., Bermejo-Martin J.F., Ferrer R., Riera J. (2023). Procalcitonin and C-reactive protein to rule out early bacterial coinfection in COVID-19 critically ill patients. Intensive Care Med..

[26] DE Backer D., Creteur J., Zhang H., Norrenberg M., Vincent J.-L. (1997). Lactate production by the lungs in acute lung injury. Am. J. Respir. Crit. Care Med..

[27] Gershengorn H.B., Patel S., Shukla B., Warde P.R., Soorus S.M., Holt G.E., Kett D.H., Parekh D.J., Ferreira T. (2021). Predictive Value of Sequential Organ Failure Assessment Score across Patients with and without COVID-19 Infection. Ann. Am. Thorac. Soc..

[28] Huber M., Bello C., Schober P., Filipovic M.G., Luedi M.M. (2024). Decision Curve Analysis of In-Hospital Mortality Prediction Models: The Relative Value of Pre- and Intraoperative Data for Decision-Making. Anesth. Analg..

[29] Riley R.D., Hayden J.A., Steyerberg E.W., Moons K.G.M., Abrams K., Kyzas P.A., Malats N., Briggs A., Schroter S., Altman D.G. (2013). Prognosis Research Strategy (PROGRESS) 2: Prognostic factor research. PLoS Med..

[30] Steyerberg E.W., Moons K.G., van der Windt D.A., Hayden J.A., Perel P., Schroter S., Riley R.D., Hemingway H., Altman D.G., PROGRESS Group (2013). Prognosis Research Strategy (PROGRESS) 3: Prognostic model research. PLoS Med..

[31] Hingorani A.D., Windt D.A.V.D., Riley R.D., Abrams K., Moons K.G.M., Steyerberg E.W., Schroter S., Sauerbrei W., Altman D.G., Hemingway H. (2013). Prognosis research strategy (PROGRESS) 4: Stratified medicine research. BMJ.

