# Supplementary material for: Predictive Performance of SAPS-3, SOFA Score, and Procalcitonin for Hospital Mortality in COVID-19 Viral Sepsis: A Cohort Study
Source: Life (Basel). 2025 Jul 23;15(8):1161. doi: 10.3390/life15081161 (PMC12387343; doi:10.3390/life15081161)
Supplement: Supplementary file 1 [file life-15-01161-s001.zip › life-3731316-supplementary.pdf]

Supplemental File

Summary

FIGURE S1: 3

FIGURE S2: 3

FIGURE S3: 4

FIGURE S4: 4

FIGURE S5: 5

FIGURE S6: 5

FIGURE S7: 6

TABLE S1: 7

TABLE S2: 8

Figure S1: Lactate histogram stratified by hospital mortality

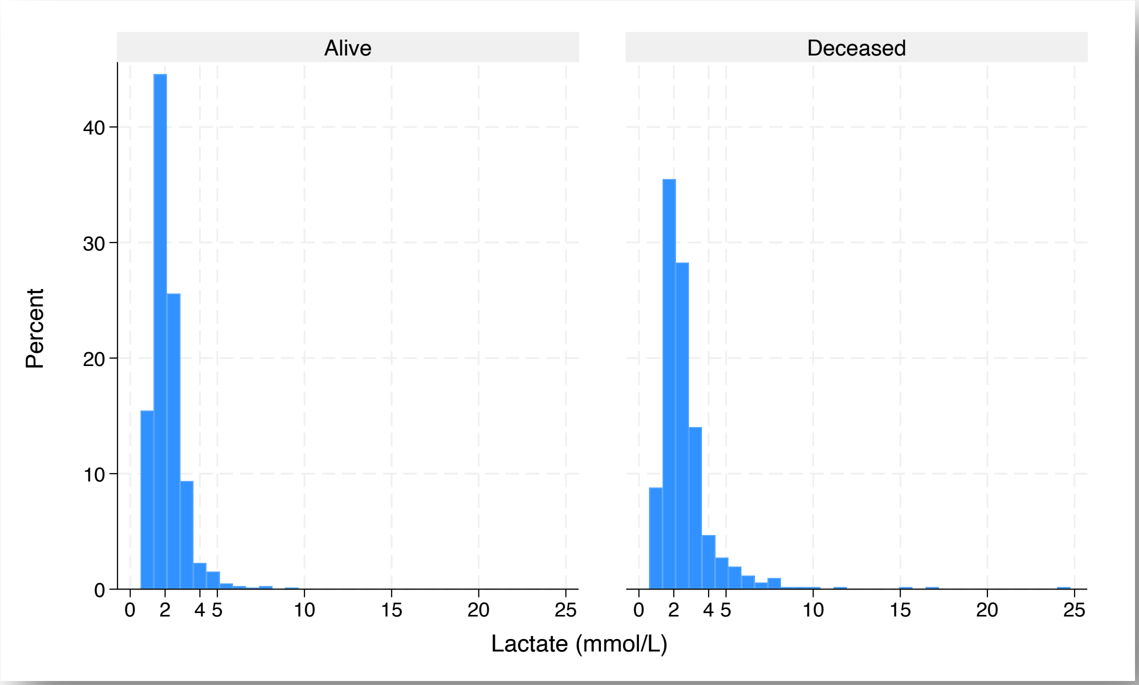

Figure S2: Lactate dehydrogenase histogram

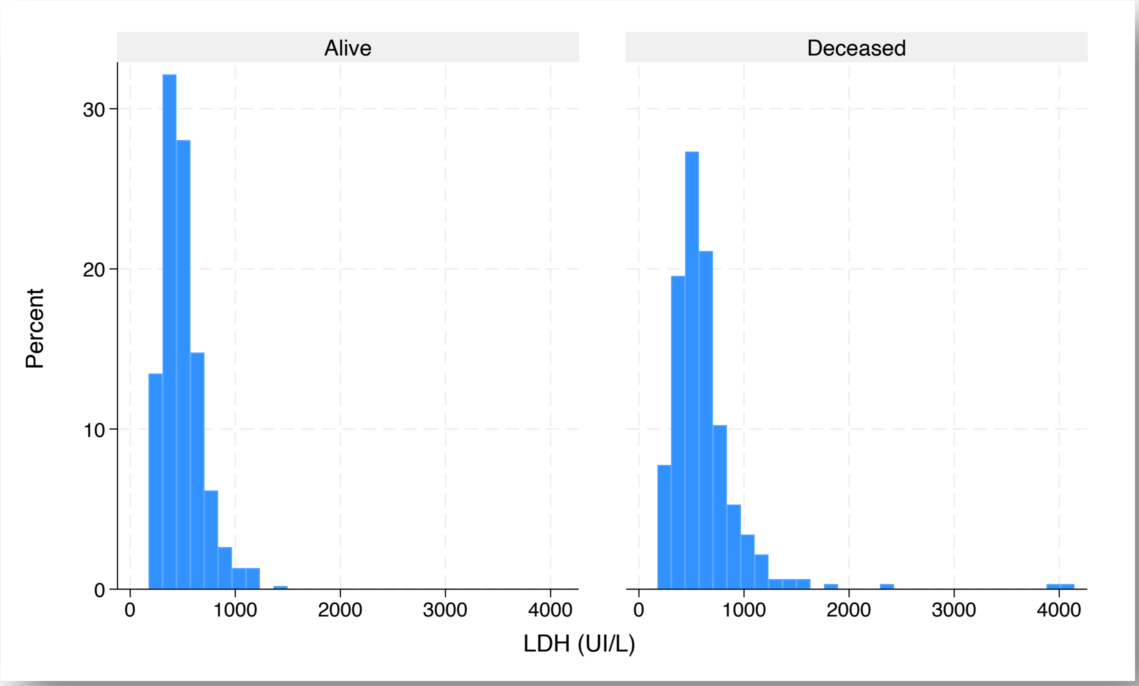

Figure S3: C-reactive protein histogram

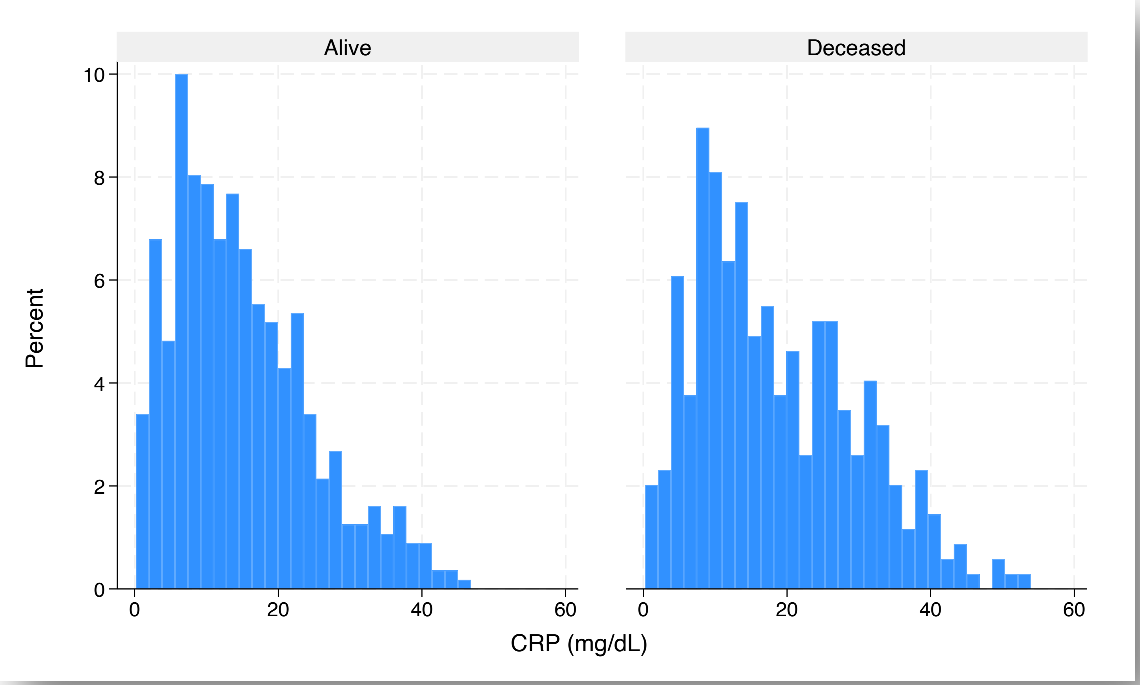

Figure S4: Procalcitonin histogram

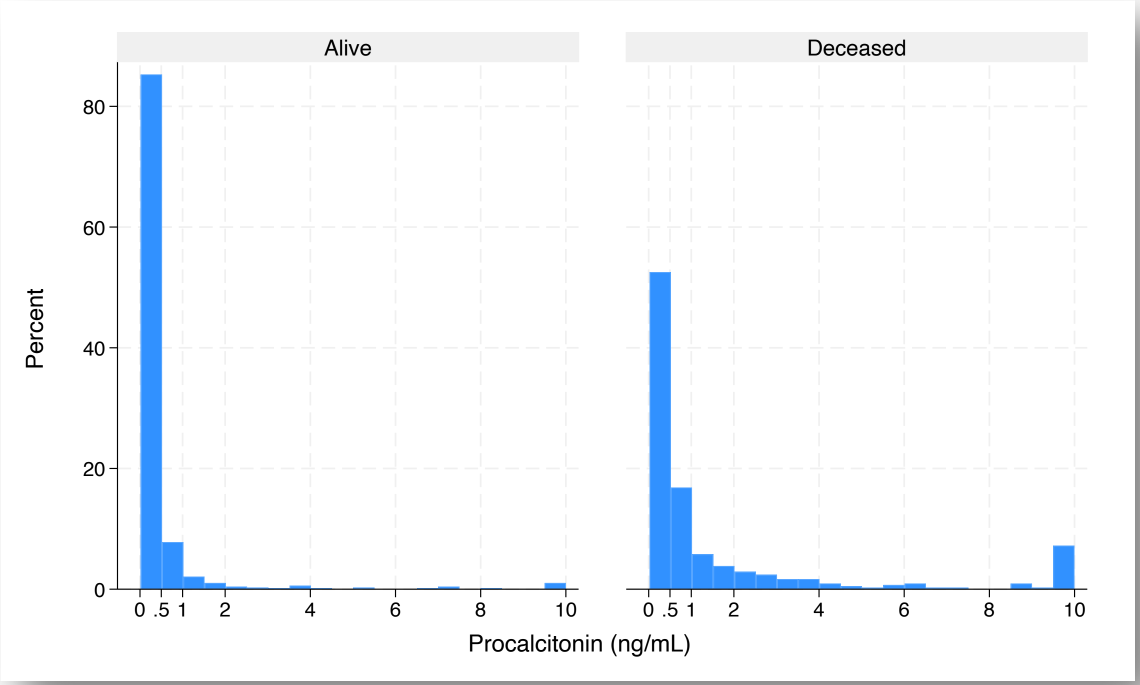

Some procalcitonin values (35) were truncated at 10 ng/mL

Figure S5: Admission SOFA score histogram stratified by hospital mortality

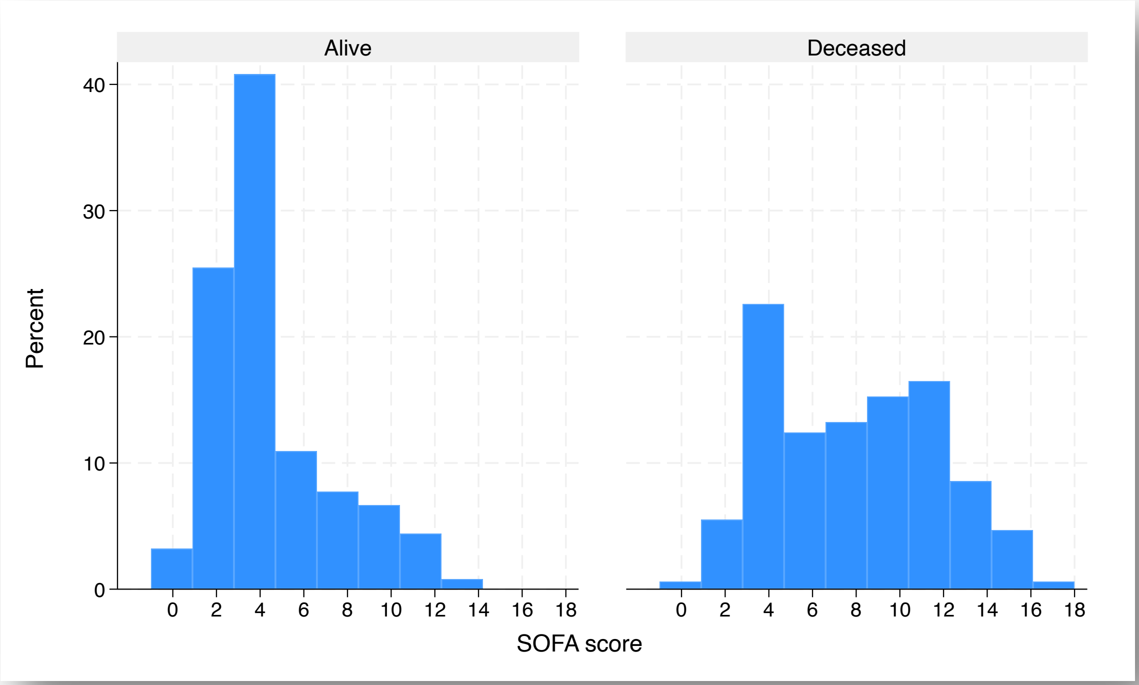

SOFA: sequential organ failure assessment

Figure S6: Bar plot of risk of hospital death stratified by admission SOFA score

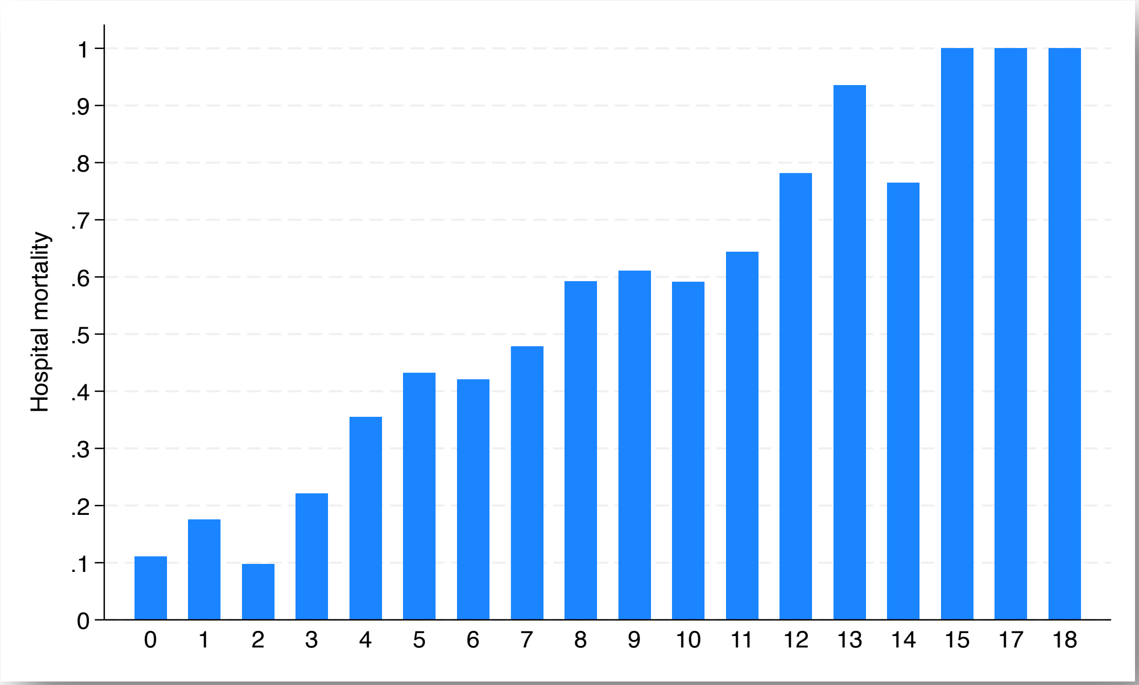

Figure S7: Apparent calibration for SAPS 3, SOFA score and other combinations of variables

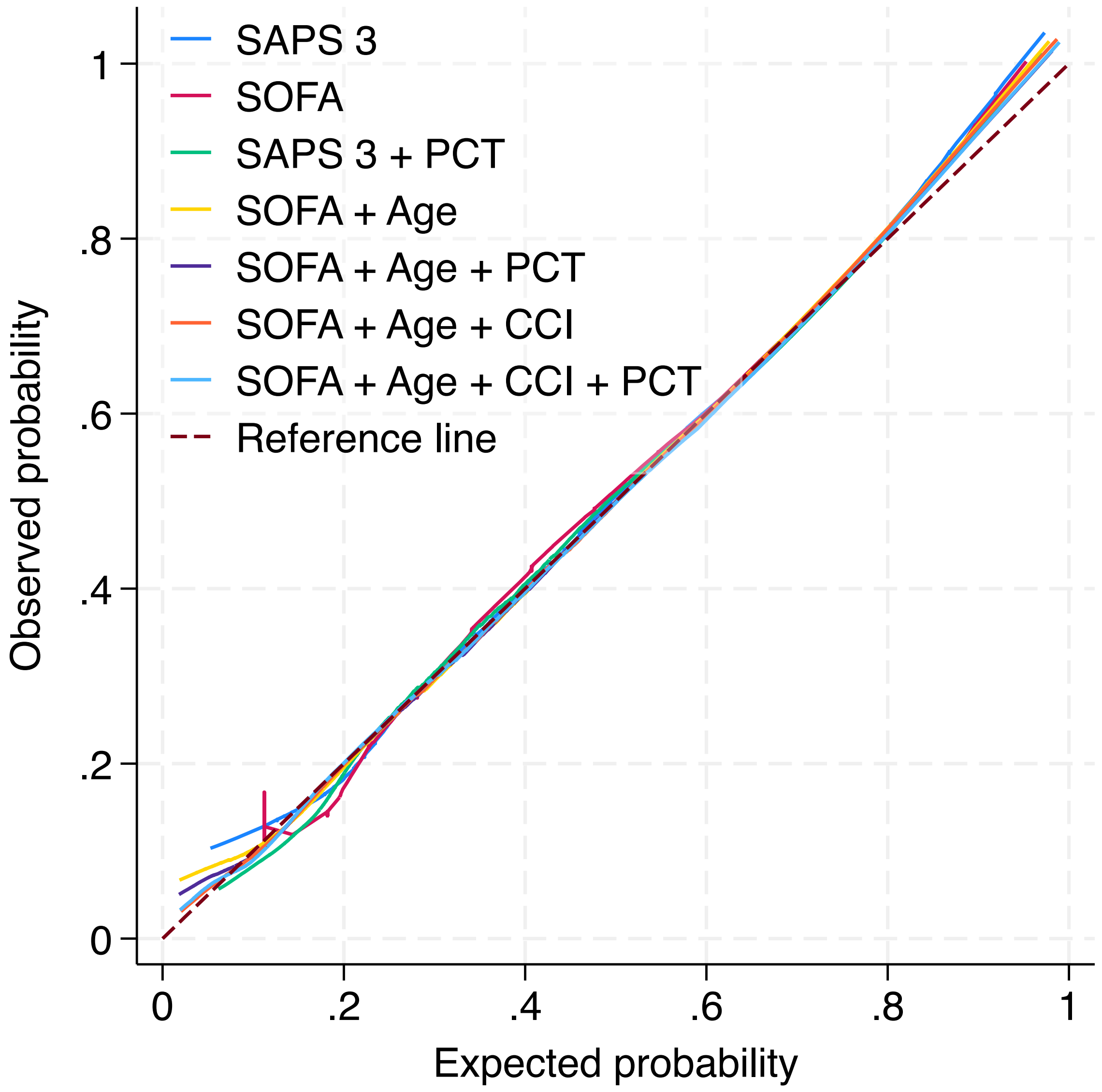

Table S1: Comparison of characteristics between patients with and without missing data*

Variable	No Missing data<br>N = 379	With Missing data<br>N = 1016	p-value
Age, mean (SD)	54.4 (16.1)	55.0 (16.2)	0.55
Male sex, n (%)	232 (61.2%)	576 (56.7%)	0.14
CCI, median (IQR)	0 [0, 2]	0 [0, 2] (n=724)	0.53
SAPS 3, mean (SD)	58.0 (16.6)	57.6 (16.4) (n=883)	0.64
SOFA D1, median (IQR)	4 [3, 9]	4 [3, 8] (n=862)	0.75
SOFA D2, median (IQR)	4 [3, 10] (n=367)	5 [3, 9] (n=887)	0.67
SOFA D3, median (IQR)	5 [3, 10] (n=176)	6 [3, 9] (n=571)	0.87
Lactate, median (IQR)	2.1 [1.6, 2.7]	2.1 [1.6, 2.7] (n=924)	0.39
CRP, median (IQR)	14.8 [8.6, 24.1]	14.0 [8.2, 22.6] (n=527)	0.21
PCT, median (IQR)	0.2 [0.1, 0.5]	0.2 [0.1, 0.7] (n=703)	0.82
LDH, median (IQR)	500 [383, 629]	488 [375, 642] (n=478)	0.38
RRT, n (%)	61 (16.1%)	174 (17.1%)<br>Missing = 22 (2.2%)	0.58
MV, n (%)	253 (66.8%)	667 (65.6%)	0.75
MV duration, median (IQR)	6 [0, 13]	5 [0, 13]	0.31
Vasoactive drug, n (%)	210 (55.4%)	622 (61.2%)<br>Missing = 2 (0.2%)	0.049
ICU LOS, median (IQR)	12 [5, 20]	11 [5, 19]	0.59
Hospital LOS, median (IQR)	16 [9, 25]	15 [9, 26]	0.98
Hospital mortality	151 (39.8%)	403 (39.7%)	0.95

* Data missingness was defined as a variable missing for SOFA (D1), SAPS 3, age, sex, CCI, RRT, MV, vasoactive drug or the biomarkers (CRP, lactate, PCT, LDH)

CCI: Charlson comorbidity index; SAPS 3: Simplified acute physiology score; SOFA: Sequential organ failure assessment; CRP: C-reactive protein; PCT: procalcitonin; DHL: Lactate dehydrogenase; RRT: renal replacement therapy; MV: mechanical ventilation; ICU: intensive care unit; LOS: length-of-stay; SD: standard deviation; IQR: Interquartile range

Table S2: p-values of the non-linear association (restricted cubic splines) of lactate, CRP, PCT and LDH with hospital mortality after accounting for illness severity (SAPS 3) or relevant covariates (SOFA, age and CCI)

	Model 1: Unadjusted	Model 2: Adjusted for other biomarkers	Model 3: Further Adjusted for SAPS 3	Model 4: Further adjusted for SOFA, age and CCI
Lactate	< 0.001	0.026	0.56	0.53
C-reactive protein	0.014	0.89	0.82	0.82
Procalcitonin	< 0.001	< 0.001	< 0.001	< 0.001
Lactate dehydrogenase	< 0.001	0.34	0.43	0.43

Results obtained in the multiply imputed datasets as described in the main manuscript.
